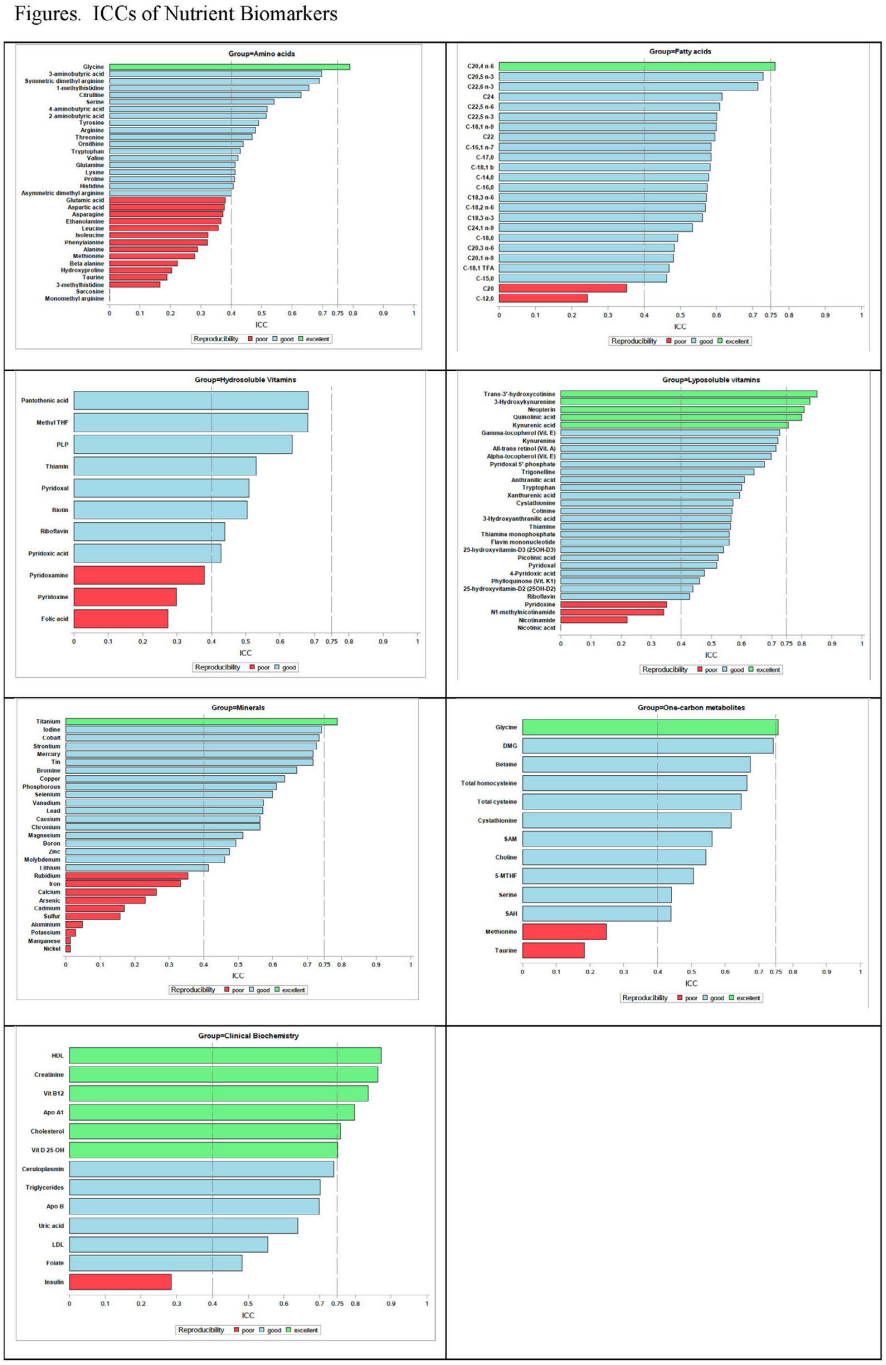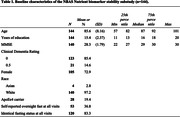# Comprehensive nutrient biomarker assessment in older adults at risk for dementia: Pre‐analytical and intra‐individual stability over 2‐4 years in the Nutrition and Brain Aging Study

**DOI:** 10.1002/alz.093020

**Published:** 2025-01-09

**Authors:** Gene L. Bowman, Jonathan A Duskin, Juliana A Haber, Nora Mattek, Hira Shrestha, John Corthésy, Hiroko H Dodge, Lisa C Silbert, Joseph F Quinn, Jeffrey A Kaye

**Affiliations:** ^1^ McCance Center for Brain Health, Department of Neurology, Massachusetts General Hospital and Harvard Medical School, Boston, MA USA; ^2^ Edacious, PBC, Marborough, MA USA; ^3^ NIA‐Layton Aging & Alzheimer’s Disease Research Center, Portland, OR USA; ^4^ McCance Center for Brain Health, Massachusetts General Hospital, Boston, MA USA; ^5^ Nestlé Institute of Health Sciences, Lausanne Switzerland; ^6^ Massachusetts General Hospital, Harvard Medical School, Boston, MA USA; ^7^ NIA‐Layton Oregon Alzheimer’s Disease Research Center, Oregon Health & Science University, Portland, OR USA; ^8^ VA Portland Healthcare System, Portland, OR USA; ^9^ Oregon Health & Science University, Portland, OR USA

## Abstract

**Background:**

Nutrient biomarkers (NBs) may serve as more accurate and precise indicators of dietary intake, particularly in older adults who often have subtle episodic memory recall and digestive/microbiome issues that alter the nutritional substrate readily available to the brain. NBs also allow insight into modes of action and metabolic changes that are actionable targets for modification through nutritional means in hopes of disease prevention and treatment. Prior to broad investment and deployment, the pre‐analytical and intra‐individual temporal variation over time should be documented to design and interpret clinical and epidemiological studies properly. We quantified 155 NBs and calculated their intra‐individual stability over time as selection criterion for future research to establish nutritional requirements for brain health and dementia prevention.

**Method:**

Blood samples from three time points, spanning a 2‐4 years, were obtained from older adults participating in the NIA‐Layton Oregon Alzheimer’s Disease Research Center’s Nutrition and Brain Aging Study (NBAS). Blood samples were batched randomly across time points for quantification of blood amino acids, minerals, hydro‐and‐liposoluble micronutrients, lipids, 1‐carbon, and kynurenine pathway metabolites using a variety of state‐of‐the‐art methods including, inductively coupled mass spectrometry. Pre‐analytical coefficients of variation (CV) and intraclass correlation coefficients (ICC) were calculated for each NB to evaluate the intra‐individual stability in a subset of NBAS cohort (n = 315).

**Result:**

The mean analytic sample (n = 137) baseline age was 85.6 (±8.3, 57‐101 years), and mean MMSE was 28.3 (±1.78) with all having Clinical Dementia Rating of 0.5 or less. Seventy‐three percent were female and 19% carried the apoEe4 allele. The pre‐analytical CVs range from 0.9 to 55.0% and the ICC from 0 to 0.87 (25%‐tile/***median***/75%‐tile 0.41/***0.54***/0.66). Twenty four percent had ICC< 0.40, 66% had ICC 0.40‐0.75 and 10% had ICC>0.75.

**Conclusion:**

The pre‐analytical and intraindividual variation of NBs ranges widely in older adults. Most can reasonably estimate average blood concentrations over a 4‐year period using a single measurement (ICC≥0.40). NBs with ICC above 0.40 can be used for measurement error correction and those with ICC< 0.40 require repeat sampling and further investigation into the methodological/biological source of variation over time in older adults at risk of dementia.